# Guillain-Barré syndrome, tuberculosis and inflammatory bowel disease: a multiple association

**DOI:** 10.1186/1755-7682-3-15

**Published:** 2010-07-16

**Authors:** Ricardo Gómez de la Torre, Germán Morís, Desireé Pérez Martínez, Isabel Carrio Montes

**Affiliations:** 1 Internal Medicine Service. Hospital San Agustín, Avilés, Asturias, Spain; 2 Neurology Service. Hospital San Agustín, Avilés, Asturias, Spain

## Abstract

Guillain-Barré syndrome (GBS) has been associated with both infective or non-infective aetiologies. GBS is usually preceded by acute respiratory or gastrointestinal infection but its association with tuberculosis has been exceptionally reported. Inflammatory bowel disease (IBD) is associated with clinical manifestations involving the neurological system, peripheral neuropathy is known to be related to IBD and, either demyelinating or axonal involvement of peripheral nerves have been described. We report an unusual case of GBS associated with lymph node tuberculosis and ulcerative colitis.

## Background

Guillain-Barré syndrome (GBS) is an acquired inflammatory peripheral neuropathy defined by acute onset, cerebrospinal fluid (CSF) albumin cytological dissociation and a clinical monophasic course with partial or total recovery. Most evidences support the hypothesis of an autoimmune origin, with cellular and humoral immunity working together against an aggressive agent. In 60% of cases, the onset is preceded by acute respiratory or gastrointestinal tract infection, although in most of the cases it is not possible to know the causal agent [1].

Peripheral neuropathy is known to be related to inflammatory bowel disorders (IBD) and is one of the most frequently reported neurologic complications. Demyelinating or axonal involvement of peripheral nerves in IBD have been described, and both neuropathies could be acute or chronic. On the one hand, acute and chronic demyelinating inflammatory polyneuropathy has been reported, as well as multifocal motor neuropathy; on the other hand, mononeuritis multiple and large- and small-fibre axonal neuropathy have been also found [2-5].

Neuropathies in association with tuberculosis (TB) infections are uncommon but have been previously reported to occur due to associated malnutrition, alcoholism, and the neuropathic effects of medication [6]. However, association of GBS and TB has been reported [7-11], moreover, in a recent report, Soysal y Cols., found 2 out of 104 cases of GBS to be associated with TB [12].

We report an unusual case of GBS with a multiple association. The patient was diagnosed with lymph node tuberculosis (LNT) and suffered GBS early after treatment onset. Later, in the evolutionary course he presented an outbreak of ulcerative colitis (UC) previously unknown. In our knowledge, this very unusual association has never been described.

## Case Presentation

A 74 year old man was seen as an outpatient in the Haematology Division after finding macrocytosis on a usual complete blood count (CBC). His previous clinical history had been unremarkable. During the physical examination, vital signs were normal, cervical adenopathy was palpable and body mass index was 20 kg/m^2^. A CBC test showed monocytosis, reticulocyte count was normal and direct Coombs was negative. Bone marrow aspirate was hypercellular with appropriate haematopoietic responsiveness without dysplastic expression and a normal myeloerythroid ratio; in erythroid series giant forms were observed; in myeloid series cytoplasmatic degranulation and pseudo Pelger-Huet forms with low increase in myelocites and moderate increase of monocytes count were found; no cellular niches were seen. Biochemical test panel was irrelevant. Serological tests for cytomegalovirus, toxoplasmosis, syphilis, Epstein-Barr virus, human immunodeficiency virus and type A, B & C hepatitis virus were negative. The tuberculin test was reactive in 5 mm. The cervical fine needle aspirate was negative for malignancy and diagnosed for lymphadenitis, a pathological specimen showed extensive necrotic inflammation with caseum aspect; microscopic examination revealed multiple epithelioid cells, granulomas and multinucleated giant cells, and extensive central necrosis; chronic lymphocytic infiltrate and fibrosis, plasma cells and histiocytes were seen in the periphery. These features were related to the diagnosis of LNT. Sputum and urine smears were negative in two results; lymph node culture was positive for mycobacterium tuberculosis, with normal susceptibility to usual drugs. No abnormalities were depicted in chest radiograph. The patient was given isoniazid (300 mg daily), rifampicin (600 mg daily), and pyrazinamide (1200 mg daily).

Six days later, the patient suffered acute and progressive legs weakness that ascended to the arms in the first 24 hours, without sensory loss. A lumbar puncture showed normal protein at 0.36 g/L without pleocytosis. CSF smear was normal. Genomic study and culture for mycobacterium tuberculosis were negative as well as cytological CSF. Cranial computer tomography showed non-specific brain atrophy. Neurophysiological study was consistent with motor axonal polyneuropathy without evidence of acute denervation (Table 1). Antibodies to nuclear antigens were negative, as well as genomic test for Lyme and Campylobacter jejuni. Urine levels of uro/coproporphyrins were not increased. Antibodies binding gangliosides IgM class GM-1, GDA-1 and GM-2 were positive. Intravenous immunoglobulin therapy was given as 0.4 g/kg over 5 days, with slow neurological recovery. Two months later the patient suffered an enteric diarrhoea with bloody stool, fever and abdominal pain. Stool cultures for bacterial pathogens and serologic tests for amebiasis were negative. An endoscopic view of the colon showed friable mucosa, extensive ulceration and exudates. Pathological findings at colon and rectum biopsies consisted of neutrophils in mucosa and submucosa, neutrophil clumps in crypt lumen (crypt abscesses), mucosal edema and vascular congestion with focal haemorrhage. Aminosaclicylates were given to the patient who experienced partial clinical improvement.

**Table 1:** Patient neurophysiological data.

RMN			LMN			RUN		
	**RV**	**NV**		**RV**	**NV**		**RV**	**NV**

**DL**	4.4 ms	> 4.6 ms	**DL**	4.1 ms	< 4.6 ms	**DL**	5.9 ms	< 4.2 ms

**MAP**	2 mV	> 8 mV	**MAP**	1 mV	> 8 mV	**MAP**	1.5 mV	> 7 mV

**SCV**	53 m/s	> 43 m/s	**SCV**	51 m/s	> 43 m/s	**SCV**	45 m/s	> 45 m/s

**SAP**	5 μV	> 36 μV	**SAP**	8 μV	> 36 μV	**SAP**	12 μV	> 32 μV

**LR**	ND							

								

**EMG**								

	RAPB N						RADM N	

RMN: right median nerve; LMN: left median nerve; RUN: right ulnar nerve; RV: recorded values; NV: normal values; DL: distal latency; MAP: muscular amplitude potential; SCV: sensory conduction velocity; SAP: sensory amplitude potential; LR: late responses; ND: not detectable; EMG: electromyographic features; RAPB: right abductor pollicis brevis muscle; RADM: right abductor digiti minimi; N: no pathological neurogenic features.

## Discussion

Peripheral neuropathies in association with TB infection are very uncommon reported except polyneuropathy related to isoniazid and ethanbutol toxicity, apart from these toxic forms, reports are in form of acute or chronic demyelinizanting polyneuropathy [6]. The exact pathogenesis of SGB associated with TB remains obscure but it is believed to be due to a molecular mimicry leading to the immunological attack of peripheral nerves. This concept of molecular mimicry is further supported by studies showing clinical improvement after receiving immunomodulatory agents. In addition, direct involvement of the peripheral nervous system has been reported as a result of meningeal involvement, direct nerve root involvement, and granuloma affecting peripheral nerves [8-10].

Although UC and Crohn's disease (CD) have been traditionally considered limited to the gastrointestinal tract, it is known that numerous extraintestinal manifestations (EM) affect many systems. Prevalence is high, EM related with IBD appeared at least once in 46.6% of the patients. EM are lightly more frequent in CD than UC and their rate increases with their evolution. Although rare, neurological complications can be very serious and irreversible without an initial appropriate treatment. Little is known about neurological EM prevalence, but 20-30% has been reported, some cases preceding IBD and some being diagnosed after their remission [13-15]. Several pathogenic mechanisms have been suggested to explain the affectation of peripheral and central nervous system by IBD: malabsorption, deficiency of vitamins, formations of toxic agents, direct effects of the treatment (infections, complications of the immunosuppression), thromboembolism disease and immunological problems [13,14]. Peripheral neuropathies related to IBD seems to be more frequent in UC, with a reported incidence of 1,9%. However, it seems that UC presents a lower rate of demyelinating forms as compared to CD. The treatment of these patients is controversial but some cases of good evolution are described with corticosteroids and 5-aminosalicylic acid [13-16].

The neurophysiological study of our patient showed an acute axonal motor neuropathy subtype of GBS in spite of normal CSF protein level because elevated CSF protein may not be apparent in the first 7 to 10 days of the acute illness in up to 10% cases of GBS [1]. GBS developed after the treatment of TB started, therefore GBS might be associated with TB rather than UC, although disturbances in the immune system due to UC played a role on the development of GBS. This neurological complication is interpreted in terms of a paradoxical disease expansion during antituberculous therapy without drug resistance [10]. This situation appears after initiating treatment in a local expansion form or in a systemic dissemination to other systems, mainly nervous system and skin. The antigens production mechanism would be caused by the liberation of tuberculosis bacillus proteins or by the liberation of cytokines, mainly tumour necrosis factor alpha, both related to the action of tuberculosis treatment [10].

## Conclusion

We present a case of GBS occurring in association with LNT and IBD. Descriptions of association between TB and GBS have been reported but this unique disorder combination has seldom been described. We suggest that GBS may be related to the preexisting chronic IBD and may be regarded as a possible EM of UC. As some patients with GBS also present an infectious antecedent, the coincidental LNT may play a role in the pathogenesis of GBS. Our case highlights the need to considerer TB in patients with GBS.

## Consent

Written informed consent was obtained from the patient for the publication of the Case Report. A copy of the written consent is available for review by the Editor-in-Chief of this Journal.

## Competing interests

The authors declare that they have no competing interests.

## Authors' contributions

RGT, GM, ICM and DP contributed to the discussion and the writing of the manuscript.

All authors read and approved the final manuscript

## References

[B1] Hughes RA Cornblath DR Guillain-Barré syndrome Lancet 2005 366 1653 1666 10.1016/S0140-6736(05)67665-9 16271648

[B2] Higelmo MA Barrio J Cosme A Isturiz JJ Guillain-Barré syndrome in a patient with ulcerative colitis in remission Gastroenterol Hepatol 1999 22 114 10193098

[B3] Gondim FAA Brannagan TH Sander H Chin RL Latov N Peripheral neuropathy in patients with inflammatory bowel disease Brain 2005 128 867 879 10.1093/brain/awh429 15705608

[B4] Oliveira GR Teles BC Brasil EF Souza MH Furtado LE de Castro-Costa CM Rola FH Braga LL de A Gondim F Peripheral neuropathy and neurological disorders in an unselected Brazilian population-based cohort of IBD patients Inflamm Bowel Dis 2008 14 389 395 10.1002/ibd.20304 17924556

[B5] Sassi SB Kallel L Ben Romdhane S Boubaker J Filali A Hentati F Peripheral neuropathy in inflammatory bowel disease patients: a prospective cohort study Scand J Gastroenterol 2009 44 1268 1269 10.1080/00365520903199871 19681018

[B6] Shin SS Hyson AM Castañeda C Sánchez E Alcántara F Mitnick CD Fawzi MC Bayona J Farmer PE Kim JY Furin JJ Peripheral neuropathy associated with treatment for multidrug-resistant tuberculosis Int J Tuberc Lung Dis 2003 7 347 353 12729340

[B7] Vyravanathan S Senanayake N Guillain-Barré syndrome associated with tuberculosis Postgrad Med J 1983 59 516 517 10.1136/pgmj.59.694.516 6622344 PMC2417601

[B8] Chong VH Joseph TP Telisinghe PU Jalihal A Chronic Inflammatory demyelininating polineuropathy associated with intestinal tuberculosis J Microbiol Immunol Infect 2007 40 377 80 17712474

[B9] Soehard YZ Yuhanissa A Thein SS Rohana AG Fauzi AR Norlinah MI AMSAN variant of Guillain-Barré syndrome progressing to Chronic Inflammatory Demyelinating Pollyneuropathy in a patient with Marfan's syndrome and pulmonary tuberculosis Med J Malaysia 2005 60 655 656 16515122

[B10] Fernández-Fúnez A Gómez Garrido J Alamillo A Sáez L Demyelinating polyneuropathy as the Honest form of lymph node tuberculosis. Paradox response in an immunocompetent patient Med Clin 2007 129 78 79 10.1157/13106942 17588368

[B11] Sabriá Mestres J Alijotas Reig J Ordi Ros J Biosca Gómez de Tejada M Sumalla Suñé J Guillain-Barré syndrome and tuberculosis. A chance association? Rev Clin Esp 1985 176 320 321 4001495

[B12] Soysal A Aysal F Calıskan B Dogan Ak P Mutluay B Sakallı N Baybas S Arpacı B Clinico-electrophysiological findings and prognosis of Guillain-Barre syndrome-10 years' experience Acta Neurol Scand 2010 in press 20497128 10.1111/j.1600-0404.2010.01366.x

[B13] Roca B Moreno I Meneu E Ulcerative colitis and acquired demyelinating neurophaty (Guillain-Barré syndrome) Neth J Med 1999 54 129 130 10.1016/S0300-2977(98)00147-8 10189787

[B14] El Moutawakil B Rafai MA Gam I Tahiri JM Rhimou A Slassi I Crohn's disease presenting with reccurent acute polyradiculoneuropathy Rev Neurol 2007 163 244 246 10.1016/S0035-3787(07)90398-7 17351546

[B15] Krystallis CS Kamberoglou DK Cheilakos GB Maltezou MN Tzias VD Guillain-Barré syndrome during a relapse of ulcerative colitis: a case report Inflamm Bowel Dis 2010 16 555 556 19714764 10.1002/ibd.21071

[B16] de la Fuente Fernández R Rubio-Nazabal E de la Iglesia Martínez F Guillain-Barré syndrome as an extraintestinal manifestation of Crohn's disease Postgrad Med J 1995 71 437 438 10.1136/pgmj.71.837.437 7567741 PMC2397986

